# Collectanea

**Published:** 1828-09

**Authors:** 


### COLLECTANEA.

Floriferis ut apes in saltibus omnia libant,
Omnia nos, itidem, depascimur aurea dicta.

### PATHOLOGY.

*Case of Rupture of the Stomach produced by Vomiting; with Observations.* By J. N. Weekes, M.R.C.S. and House Surgeon at St. Bartholomew's Hospital.—G. Andover, æt. thirty-four, had been liable for about two years to paroxysms of pain in the stomach. The pain usually continued for several hours, and generally went off with vomiting; and it returned at uncertain intervals, frequently of many weeks. Between the attacks, the patient enjoyed tolerably good health. About Christmas last, he vomited a large quantity of blood, which rendered him so feeble that he was confined to his bed for five weeks. Since that time his health has been much impaired, and the attacks of pain, followed by vomiting, have been more frequent.

On the evening of April 13th, he was brought to St. Bartholomew's Hospital, where I first saw him. He was then suffering great pain, extending from the epigastric region over the whole abdomen, and accompanied by nausea; there was neither tenderness nor tension of the abdomen; the pulse was frequent, tongue clean. He had shortly before his admission drunk some shrub-and-water, to which he in a great measure attributed these symptoms, and told me he had had a similar attack a week ago, after indulging in spirituous liquors, and that it went off with vomiting. On the following day, the pain had subsided; there had been no vomiting, but he complained of nausea; the abdomen was distended by flatus, and he had frequent eructations; the pulse was weak, tongue natural.

At eleven o'clock P.M. he had a sudden attack of most severe pain. I was called to him about an hour afterwards, and found him groaning with agony at the pit of the stomach; the abdominal muscles were hard and contracted; the belly was neither painful nor tender on pressure; his pulse was small and feeble; he was extremely restless, and his countenance expressive of the greatest suffering. I instantly gave him sixty drops of tincture of opium; and, as he found no relief, they were repeated, but without benefit. He continued to suffer most acute pain for about two hours, when he was suddenly seized with violent vomiting. After this, the pain somewhat abated; there was no return of vomiting; but he sunk rapidly, and died at four o'clock in the morning.

*Examination.*—On opening the abdomen, the stomach was observed to be flaccid and empty, and its contents, which consisted of a large quantity of dark-brown fluid, were effused into the peritoneal cavity, through a ragged opening situated on its anterior surface, and near the œsophageal orifice. The rupture extended from below the lesser arch of the stomach to near its cardiac extremity, and was about four inches in length. The three membranes were not torn equally, the rupture of the peritoneal covering extending an inch farther than that of the muscular or mucous coat. On the posterior surface of the stomach was a laceration, measuring three inches in length; and there were two or three small ones, from an inch to an inch and a half in length, at its great arch. These lacerations extended only through the peritoneal coat of the stomach, the muscular and mucous tunics remaining perfectly whole. The mucous membrane of the stomach was lined with a great deal of dark-coloured secretion, beneath which the membrane itself was of a deep red colour throughout; its texture was softened and partially emphysematous. The stomach in other respects appeared healthy; the liver was pale and softened; the gall-bladder contained a calculus; the structure of the spleen was unusually soft. The other viscera were healthy.

The most remarkable feature in the preceding case is the extensive rupture of the stomach, with so little disease of its coats; and in this respect it forms a striking difference to those cases hitherto related. The stomach presented no thickening nor ulceration at the part which was ruptured; the disease was confined to its mucous tunic, and appeared to be recent inflammation and softening of its texture. It may also be remarked, that the symptoms in this case were not such as generally indicate the existence of organic disease: there were considerable intermissions of the symptoms, the patient had enjoyed tolerably good health, and there was no emaciation.

The only case I have met with of rupture of the coats of the stomach, produced by an act of vomiting, or rather attempting to vomit, is recorded by Lallemand, and is described in the 49th volume of the Dictionnaire des Sciences Médicales, art. *Rupture.*

The patient had laboured under difficult digestion for five or six months, and had been much relieved by observing a strict regimen. After indulging her appetite to a greater extent than usual, she was attacked with uneasy feelings in the stomach, accompanied by nausea and inclination to vomit. She made violent but ineffectual efforts to discharge the contents of the stomach, and, whilst suffering great agony, experienced intense pain, with a sense of tearing at the lower part of the belly: she uttered several screams, and fell down insensible. She sunk rapidly, and died in the night. On dissection, the cavity of the peritoneum was found full of half-digested food; the anterior and middle part of the stomach was torn obliquely from its small towards its great curvature, to the extent of five inches. The edges of the rupture were thin, irregular, and presented no marks of disease. The three coats of the stomach were not torn to an equal extent, nor exactly in the same direction: the rupture in the peritoneal was larger than the muscular coat, and the mucous membrane was the least extensively lacerated. A mass of scirrhus, an inch and a half in extent, surrounded the pylorus. The other parts of the stomach were perfectly healthy.—*Medico-Chirurg. Transactions*, vol. xiv. part ii.

***Observations on Depositions of Pus and Lymph, occurring in the Lungs and other Viscera, after Injuries of different Parts of the Body.*** **By THOMAS ROSE, Esq. M.A. Surgeon to St. George's Hospital.—The following is an abstract of Mr. ROSE's remarks upon this interesting subject.**

It has long been known to pathologists and surgeons that abscesses occasionally occur in some of the principal viscera of the thorax and abdomen, in consequence of injuries of the head; and that, from the same cause, purulent effusions sometimes take place into the cavities of the pleura and peritoneum. This fact did not escape the notice of MORGAGNI,* who disproves, by his dissections, and by those of VASSALVA, the notion, that had been entertained by MARCHETTI, of the matter descending from the wound in the head into the cavity of the thorax. DESAULT considered abscess of the liver to be one of the most common effects of injuries of the head. In speaking of the erysipelas which attends wounds of the scalp, he observes, "Qu'il est rare que les symptomes deviennent violens, sans que le foie ne s'affecte, où même qu'un dépôt ne s'y forme." RICHERAND endeavours to prove that these abscesses must depend upon some injury which the liver had sustained at the time of the accident. They occur, however, under circumstances where such a supposition cannot possibly be entertained. It is curious that Mr. POTT is entirely silent upon this subject. Granting that abscesses of the liver, under such circumstances, may not be near so common as Desault would lead us to suppose, yet their occurrence in that as well as in other viscera, after injuries of the head, appears to Mr. Rose to have been too little considered by English surgeons. It is not after injuries of the head alone that such abscesses are formed. During the Peninsular war, Mr. R. met with several instances, in the lungs particularly, after amputations and other wounds of the extremities. It appears that LARREY, who met with a case of this kind, at first attributed the fatal result, and disease of the viscera, to the effects of an Egyptian climate, or to fatigue and other causes. He did not imagine that they were connected with the previous wound or operation. At a subsequent period, he seems to have been aware of the true nature of such cases.

"I have seen," says Mr. Rose, "repeated instances of the disease in the lungs, in the liver, and in the spleen, and after various accidents. I have not been able to discover any peculiarity of constitution which could be regarded as predisposing to it. Many of the patients were young and healthy individuals, who, until the time when they met with the accidents, had never been affected with disease. Some of them were treated on the strictest antiphlogistic plan throughout, in consequence of the nature of the accident they had experienced. In others, (in compound fractures, for instance,) as soon as the first inflammation had subsided, means were used for supporting the strength of the system. No difference as to the formation of the internal abscesses could be observed. In all the cases which I have seen, these abscesses took place at some period between the end of the second and that of the fifth week after the accident which gave rise to them.

"The theories which ascribe their formation to injury done to the liver itself at the time of the accident, to obstruction to the entrance of the blood into the right auricle through the vena cava inferior, or to a direct communication for the transmission of matter from the head to the cavity of the

* On the Seats and Causes of Diseases, translated by Dr. ALEXANDER, vol. iii. p. 100 et seq. London, 1769.

thorax, are all obviously absurd. That of Desault, which attributes them to the disturbance of the nervous system, resulting from the injury, is probably the only explanation which can be given of their cause. They are to be classed amongst the effects of constitutional irritation arising from local injury, and are certainly striking illustrations of the irregular action in the vascular system to which that irritation may give rise. The attention of the members of our profession has lately been directed to this most important subject by the very valuable work of the president of this society,* and it is to the principles which he has so ably illustrated that I should look for an explanation of the phenomena which I am now attempting to describe."

Although constitutional disturbance, evidently referrible to an unfavorable state of the wound, has, in all the cases which Mr. Rose has seen, preceded the formation of these visceral diseases, yet a favorable change has often taken place in the wound before the symptoms of the internal abscess have begun to manifest themselves; and we are able sometimes to detect the existence of the latter, by the presence of rigors and other symptoms of suppurative fever, at a time when the wound itself is disposed to heal.

" The examination after death of those who have been affected with this disease, presents appearances which are well worthy of notice, though it is not easy to convey a correct idea of them in words. The disease consists, apparently, of depositions in the cellular texture of the affected organ, partly of a white or yellowish coloured lymph, and partly of pus. These depositions vary in size from beyond the bulk of the largest walnut to something less than a common pea. Where the lymph is most abundant, they may be described as a soft white tubercle of irregular shape, not contained in a cyst, but imbedded in the cellular substance of the part, and gradually blending with its natural structure. When pressed, some pus exudes from them. Where the pus collects in greater quantity, it is lodged in an irregular cavity, probably in the middle of some of the tubercles, and the walls of the abscess are formed of flakes of lymph. The number of these tubercles and abscesses vary in different instances, there being sometimes only one or two, and sometimes the whole viscus being filled with them. In the lungs they are chiefly formed in the parts adjacent to the pleura pulmonalis, and there is often at the same time an effusion into the cavity of that membrane of a sero-purulent fluid mixed with lymph. In the liver and spleen they are dispersed throughout the substance, sometimes shewing themselves in one or more yellowish patches, not elevated, on the convex surface of the great lobe of the former viscus, and at other times lodged in its substance. The parts adjacent to them shew evident marks of increased vascularity."

Upon the treatment of such cases, Mr. Rose has little to suggest. Our efforts must be directed, 1st, to subdue any excess of arterial action; 2dly, to quiet the disturbed state of the nervous system. When the abscesses are once formed, we shall find the truth of the observation of Desault, that they are almost invariably fatal. Four cases of the disease are given, arising from injuries to different parts of the body.

Case I.—Abscesses in the lungs, with extravasations of lymph and pus into the cavities of the pleura, after wound and amputation of the arm. The patient died on the seventh day after the operation, which was rendered neces-

* Vide an Inquiry concerning Constitutional Irritation, by Benjamin Travers, Esq. F.R.S. London, 1826.

sary by a wound he received at the storming of St. Sebastian's. In the cavity of the thorax, on the left side, more than a pint of sero-purulent fluid was found effused, mixed with loose flakes of coagulable lymph. There were several abscesses in the lungs. Abdominal viscera healthy.

In Case II. abscesses were found in the lungs, liver, and spleen, after compound fracture of the leg.

Case III. is especially interesting. We give it at length.

" *Abscesses in the Lungs, Liver, and Articulation of Clavicle and Sternum, with Effusion into the Thorax, after a Bruise and Wound of the Foot, and a fractured Fibula.*—George Stacey, eighteen years of age, and apparently of a healthy constitution, was admitted under my care into St. George's Hospital, on the 17th of July, 1827, in consequence of an accident from a cart-wheel having passed over the outside of his left foot. There was a small wound under the little toe, made apparently by some sharp substance, which had penetrated under the first phalanx, about an inch into the sole of his foot. Considerable ecchymosis had taken place over all his instep and foot, and there was a simple fracture of his left fibula two inches above the ankle. Leeches, cold lotions, and aperient medicines were ordered, and the limb was kept quiet, and supported on a pillow. The leeches were repeated several times.

On the 23d, he had shiverings, after a restless night; and these were followed by diffused cellular inflammation over every part of the foot, and by erysipelas extending up the leg and thigh, with enlarged glands in the groin. The integuments in different parts of the foot were divided, to set the inflamed parts at liberty; and, on free openings being obtained for matter which had formed under the fascia plantaris, the febrile disturbance began to subside.

" On the 4th of August, he was reported convalescent, and at his earnest request was ordered some meat for his dinner on the following day.

" On the 5th, he had a severe rigor, which lasted for more than an hour.— A purgative medicine was ordered, and he was again put on light diet; and it is to be observed that the rigor came on before he had taken the meat.

" On the 7th, the rigor returned at the same hour as on the 5th, and lasted about the same time. The limb continued perfectly quiet, all the wounds were healing, and no cause could be discerned for these febrile attacks. He had never had ague, but stated that where he had been working that disease prevailed.—He was directed to take two grains of the sulphate of quinine every four hours.

" The rigor returned again on the 8th, followed by much heat and a very quick pulse, and continued afterwards to recur at irregular intervals, being generally succeeded by profuse sweats.

" On the 10th, it was observed that he had slight ptosis of the upper eyelid of the right eye; his pulse was quick, nearly 150; his tongue dry; his countenance unfavorable, and with a yellowish tinge. There was no appearance of matter forming in any part of the leg, and he could bear pressure over the abdomen. In the evening, some degree of emphysema and a little effusion of fluid were detected at the articulation of the right clavicle with the sternum. He had met with no accident in the part to account for this.

" On the evening of the 11th he died, being the twenty-sixth day after the accident.

" The body was examined on the following day. In the head, the arachnoid appeared more opake than natural, and there was some lymph effused on the under surface of the anterior lobes of the cerebrum, and round the junction of the optic nerves; matter was found effused into the cellular membrane over the sternal extremity of the right clavicle, and into the synovial cavities on each side of the inter-articular cartilage between that bone and the sternum.

"The pleura on both sides of the thorax was very vascular, and distended with a considerable quantity of a sero-purulent fluid, mixed with loose flakes of lymph. This was more abundant on the left than on the right side of the chest.

"The lungs on each side contained numerous small abscesses and soft tubercular masses, principally adjoining the surface of the pleura. These varied in size from that of a hazel-nut to less than that of a small pea; and in the middle of some of the tubercles there was an irregular cavity filled with pus. One small abscess was found in the substance of the great lobe of the liver, at some distance from its surface. Drawings exhibiting the appearances in the lungs and liver of this man were made by an excellent artist for my friend Dr. SEYMOUR, who has been so obliging as to favor me with them to lay before the Society; and engravings from them are annexed to this volume."

In further illustration of the same subject, other cases are related, which were communicated by Mr. LAWRENCE.

Mr. GUTHRIE has made many good practical observations upon this subject, in his Treatise on Gun-shot Wounds.*—*Ibid.* part i.

---

*Cases of Tumors in the Abdomen arising from Organic Disease of the Stomach, with Remarks.* By EDWARD J. SEYMOUR, M.D. Fellow of the Royal College of Physicians, &c. &c.—From this very interesting communication we extract the following case:

Mr. C., æt. fifty-nine, a gentleman who had always enjoyed good health, and was remarkably temperate in his habits, but much occupied by anxious professional business, consulted me in the month of November 1825, being affected with pain in the region of the bladder, particularly felt after voiding his urine, which was high coloured and deposited freely uric acid.—The warm bath, and the use of soda and opium, shortly relieved these complaints; a visit to the seaside, and the moderate use of tonics, completely restored him.

About November 1826, he mentioned to me that he was occasionally troubled with water-brash, which he described as a small portion of tasteless fluid rising occasionally into his mouth, unattended by pain or any uneasiness whatever. His appetite was extremely good, sleep undisturbed; he had no pain in any part of his body. His pulse was not strong, but regular, and of natural frequency, and he described himself to be in good health.—He was recommended twenty minims of Liq. Potassæ in lime-water twice in the day; but the inconvenience appeared to have been so slight, that he did not comply with the prescription.

On the 13th March, 1827, while visiting another patient in the family, I observed that Mr. C.'s countenance and manner betrayed considerable indis-

* Third edition, p. 257 et seq.

position, and I inquired if he were suffering from return of pain in the bladder. He replied, he thought he had taken cold, and that he was much harassed by business. He said he felt as if he required opening medicine.—I ordered him an aperient, and desired he would lie in bed in the morning that I might examine his abdomen, as on pressing him through his dress there appeared some tenderness present.

14th.—The patient being in bed, the symptoms were as follow: Bowels freely open from the medicines; dejections loose, but of good colour; pulse 110, extremely weak; urine very turbid; tongue red and shining; appetite good; great sensation of debility; with an exsanguine appearance of the countenance, the less remarkable as the patient had always been unusually pale.

About midway between the umbilicus and superior anterior spinous process of the left ilium, a tumor was observed, of the size of a large orange, extremely hard, extending over about half an inch to the right side of the umbilicus, and an inch below it. This tumor was adherent to the integuments, was rather moveable, and there was considerable tenderness on pressure. Notwithstanding the size of the tumor, its tenderness, and its prominent figure, the patient, until my examination, was totally ignorant of its existence. The apparently rapid growth of the tumor, its hardness and irregularity, combined with the bloodless appearance of the patient, and the great and sudden loss of strength experienced, induced me to believe that the disease was of a malignant nature.—A dozen leeches were ordered to the part, and a consultation took place in the evening with Dr. Nevinson. Dr. Nevinson was likewise of opinion that the disease was of a malignant kind, but no decision could be formed as to which of the viscera it affected particularly.

> Hirudin. xij. tumori.—Capiat Pil. Sapon. c. Opio gr. iij. h. s.—℞. Mist. Camph. ℨx.; Sp. Æther. Nitr. ℨss.; Confect. Arom. ℈j. M. fiat haustus quartis horis sum.—Light nourishment.

15th.—A consultation took place with Mr. Brodie, who agreed in the opinion that the disease was fungous hæmatodes.

> The leeches were ordered to be repeated. Evaporating lotions to the tumor. The internal medicine to be repeated.

On the 18th, the tumor having increased, a consultation took place with Mr. Brodie and Sir A. Cooper. The latter gentleman was of opinion that the great intestine on the left side adhered to the parietes of the abdomen, that the inner coat had ulcerated, and a tumor was formed, whose contents consisted of gas, ill-conditioned matter, and feces.

> Poultices and fomentations ordered.—The soap and opium pill repeated at bedtime.—℞. Infus. Gentian. C. ℨx.; Infus. Rhei ℨij.; Pulv. Ipec. c. Opio gr. iij.; Subcarbon. Sodæ exsicc. gr. v. M. fiat haustus t. die sum.

23d.—Some fluctuation being perceived in the tumor, an opening was made to the left, a little above the umbilicus, with a lancet: about two ounces of fetid sanious pus escaped from the orifice. Some hemorrhage occurring, the pulse in the evening became extremely small and feeble; tongue red, with a brown centre; countenance much sunk; bowels purged.

> ℞. Pulv. Cret. C. ℨss.; Confect. Arom. ℈j.; T. Opii m. v.; Misturæ Camphoræ ℨx. M. fiat haustus quartis horis sumend.—Vini rubri ℥ij. ter in die.

26th.—The opening discharged freely; pulse 100; strength much improved; aphthæ in the mouth.

℞. Infus. Cuspariæ ℥x.; Confect. Arom. ℈j.; Pulv. Cretæ C. ℈j. M. fiat haustus ter die sumend.

The relief experienced by letting out the confined matter was of very short duration. The tumor enlarged as the cavity of the abscess filled up, and the condition of the patient on the 17th of April was as follows: The tumor occupies the whole of the umbilical region, being about six inches in breadth, and four in length. No pain whatever is experienced on pressure, or at any period. The cavity of the abscess filled up about one half. Bowels slightly relaxed. No vomiting or nausea. Tongue clean, less red and shining. Appetite good. Sleeps well. Pulse 100, weak.

℞. Infus. Cascarillæ ℥x.; Canell. Alb. in Pulv. ℨss.; T. Opii m. iij.; Syrupi ℨss. M. fiat haustus ter in die sum.

It now appeared expedient to endeavour, by all the means in our power, to check the growth of the tumor; and in such a case the various remedies which have been insisted on by authors for promoting the dispersion or absorption of morbid growths were fairly to be tried, however small the hopes of success which resulted from their employment.

Several blisters were applied in succession over the tumor, without affording any advantage. The tumor appeared inert, producing no pain on pressure, or during the whole process of digestion, which was uniformly to all appearance healthy, one natural evacuation being voided in the twenty-four hours; and when (which was a very rare occurrence) this was deficient, a small dose of castor-oil relieved the difficulty. The only bad symptom was the sense of extreme debility, and occasionally slight syncope.

On the 20th of May, a drachm of weak mercurial ointment was ordered to be rubbed in over the tumor daily, and three grains of bluepill given at bedtime. The cascarilla and canella, from which the patient expressed himself to derive relief, was continued. This course was persevered in for nearly three weeks, and given up without appearing to have in any way contributed to the diminution of the tumor, or the amendment of the patient's general health.

The action of iodine is at present little understood; but that it occasionally exercises very extraordinary power in the dispersion of morbid growths, is now generally admitted, at the same time that, in the present state of our knowledge, its apparent want of uniform success, and the terrible influence it exercises over the nervous system, even some weeks after its use has been discontinued, require great caution in its administration.

Half a drachm of the ointment of hydriodate of potass was rubbed in every night and morning, and five drops of the tincture given twice in the day for more than a fortnight, when the increased sense of fainting, and diminution of the patient's strength, obliged its discontinuance.

The beneficial effect occasionally produced by the internal use of the caustic alkali, especially in steatomatous tumors, suggested the propriety of employing this remedy. Twenty drops of the Liq. Potassæ were ordered to be taken thrice daily, in a little barley-water, this quantity being gradually increased to twenty-five minims five times in the twenty-four hours, which was borne without the slightest uneasiness. During three weeks that the use of this remedy was continued, a sensible amendment was perceived. Strength increased; the skin became of a healthier colour, and the tumor certainly was somewhat diminished. In consequence of this amended state, the patient left town for his seat in the country, in the middle of July. On the 1st of August he returned to London, having perceived an increase in the tumor during the preceding two days, and having experienced a return of the rising of tasteless fluid into his mouth, a symptom which had wholly left him for several months.

My attention having in the mean time been called to the case of Row, which I first detailed to the Society, I was satisfied that the malignant growth was in the stomach itself, and accordingly informed the patient's friends. This opinion was confirmed in consultation by Mr. Brodie and Dr. Chambers. After the patient returned to London, the Extr. Conii and the Liquor Arsenicalis were employed in full doses, but without any perceptibly good effect. The patient continued to decline; his hands and feet were œdematous; and his strength became so greatly impaired that he required the support of considerable quantity of stimulants, in order to maintain life and warmth.

After growing weaker and weaker through the month of September, he expired on the 2d of October without pain, having experienced a feeling of complete exhaustion, and presented an appearance of the utmost emaciation for several days previously.

The most singular circumstance attending this case, was the perfect manner in which digestion was performed during the progress of so extensive a disease of the stomach. The patient's diet consisted of broth, arrow-root, plain animal food, and white fish; and, as the disease advanced, he was permitted to drink weak brandy-and-water with his dinner, which added greatly to his comfort by counteracting the extreme sensation of debility. At no period of his disease did he experience any pain after taking food; at no period was his food returned by vomiting. The only circumstance which could draw the attention of the physician to disease of the stomach was the water-brash, but this occurred rarely in very small quantity, and was attended with no pain. The appetite continued natural until two days before death.

The body was opened twenty-seven hours after death by Mr. Brodie, assisted by Mr. Cæsar Hawkins. On the external surface of the body several spots of purpura were perceived, and a tumor was easily felt through the parietes of the abdomen, with an opening in its centre, a little above and to the left side of the umbilicus, discharging some dark purulent fluid. The cavity of the abdomen contained about three quarts of water; on the removal of which, the tumor was found to be formed by the stomach, adhering extensively to the parietes, to which the transverse part of the colon and the omentum were also joined. The stomach was opened on the posterior part, and the cardiac portion and duodenum were found to be quite healthy, the pyloric half alone being the seat of disease. It appeared to consist of a thickening of the coats of this part of the stomach, in some parts above an inch in thickness, with an irregular tumor growing from its whole circumference, of the nature of fungous hæmatodes. The whole interior surface was ulcerated, and several portions of the tumor projected into the cavity of the stomach. The tumor was soft, and highly vascular in the inner part, and gradually became firmer and whiter towards the peritoneal surface, whence several white bands ran in an irregular manner towards the interior of the tumor. The anterior part of the stomach was the thickest, particularly where it adhered to the muscles of the abdomen; and in it several abscesses were discovered, one of the largest of which was the cavity in which the opening on the surface of the abdomen terminated. The œsophagus, near its junction with the stomach, contained a small cyst of fluid, resembling an hydatid in appearance, and of the size of a filbert. The liver was rather darker than usual, but otherwise healthy, except that in the left lobe several tubercles were observed of the size of a pea, of a white colour, and of the consistence of soft cartilage. All the other viscera appeared sound.—*Ibid.*

Several cases have recently fallen under Dr. Seymour's observation, in which tumors in the abdomen, of considerable size, were found to arise from organic disease situated in the stomach. In two of them, the symptoms which have been considered to characterise organic disease of this viscus were altogether absent.

### PRACTICAL MEDICINE.

*Case of Anasarca, cured by Tartar-Emetic Ointment.*—The subject of this case was an old man, 65 years of age, of a feeble and even cachectic complexion, much addicted to drinking spirits, and obliged to work hard in the open air, and overwhelmed with grief and deprivations. After having experienced wandering rheumatic pains, for which he was treated with sudorific drinks, he was suddenly seized with general anasarca. He was very soon scarcely able to walk; his breathing became short and laborious; the weakness increased, and a slight febrile action was evident every night. The quantity of urine varied: it was sometimes abundant, and at others less than natural. The skin was always dry. The internal remedies used to excite the activity of the skin, such as the tartar emetic, acetate of ammonia, elder, arnica, &c., being unsuccessful, means which were particularly indicated by the rheumatic affection which preceded the anasarca, recourse was had to friction with tartar emetic ointment. The stomach was first rubbed, then the inferior extremities, until those parts were covered with pustules; and this treatment was continued, care being taken to keep up the excitement of the integuments by renewed frictions, in proportion as the effects of the former disappeared. At the same time tonics and diuretics were administered internally. The secretion of urine was now abundant, the patient had copious stools, and free perspirations. The quantity of urine was soon greater than that of the liquid drank.

The œdematous swelling gradually diminished from this time, first in the legs, then in the stomach, and at the end of a month all the affected parts were restored to their natural size. The pustules produced by the tartar emetic frictions discharged an enormous quantity of purulent matter.

To conclude the treatment, pills were prescribed of aloes, squills, digitalis, and cream of tartar. After a short use of them, the patient perfectly recovered, and has since remained in good health.—Graefe *and* Walther's *Journal.*

*Case of Salivation, from the external Application of Tartar Emetic.* By R. Eglesfeld Griffith, m.d.—J. M., æt. fifty, was treated for hydrothorax and œdematous swellings of the lower extremities, by active depletion, and purging with crem. tart. and jalap: no mercurial preparation was used. He was also directed to use an external application of tart. ant. ointment to his breast. This he persevered in till a large crop of pustules were produced. Shortly after their appearance his gums became sore, and a violent salivation ensued, which lasted for nearly two weeks. He used about ℥jss. of the ointment: it was made with ʒij. of tart. ant. and ℥j. axung. He is now convalescent, his disease having gradually disappeared under the salivation.

Dr. Jackson has also met with an instance in which salivation was produced by the external application of tartar emetic ointment.—*American Journal of Medical Sciences.*

---

*Eruptive Disease produced by the internal use of the Balsam Copaibæ.* (Communicated to the Kappa Lambda Society, by Thomas Hewson, M.D. &c.)—A lady had laboured under leucorrhœa for eleven years. Many physicians on the continent of Europe had been consulted, and many remedies used in vain. Equal parts of copaiba balsam and spirits of nitrous æther were given, in doses of half a drachm of the former night and morning. After having continued this medicine about a week, the patient was attacked with pains in the joints and uneasiness at the stomach, which were soon followed by itching and tingling of the skin. The next morning, she was greatly disfigured; all those parts of her body which were exposed being covered with an efflorescence of a damask-red colour. The spots, taken singly, were about three-eighths of an inch in diameter, with a slight elevation in the centre. The eyelids were swollen, and there was great swelling of the face. There was likewise considerable intumescence of the hands and arms. In the course of a few days, by omitting the copaiba, and by gentle purging, the eruption vanished.

This subject has been particularly referred to by Dr. Armstrong. Such cases are not uncommon, but they are rarely so severe as that above mentioned.

---

### SURGERY.

*Case of Extirpation of the Uterus.* By Dr. Blundell.—Mrs. A. B., æt. fifty, of grey eyes, tranquil disposition, broad in her make, and disposed to obesity, was seized with offensive discharge from the vagina, soon followed by eruptions of blood in large quantity; so that, according to her own report, frequent faintings were produced, and the blood occasionally sank through a bed about twice as thick as a sofa cushion, collecting on the floor; and day after day, for months together, with little intermission, one or two pints of blood were discharged.

Although Mrs. A. B., in her general conversation, is by no means prone to hyperbole, it seems evident that she must have greatly over-rated the quantity of these daily floodings. Certain, however, it is, from her repeated and considerate declarations, that very large quantities of blood were lost during a period of many months; and though, with the exception of some small œdema of the legs, there were no signs of general dropsy, the paleness, coldness, and weakness, and the frequent attacks of faintness, or complete delirium, shewed pretty clearly that much vascular inanition had been produced. In other particulars, the patient's condition was not altogether discouraging; for the bowels were regular, and the appetite was occasionally good; and the appearance, though cachectic, and perfectly similar to that of other women perishing under malignant ulceration of the uterus, was not such as to indicate a constitution wholly unfit for surgical operation.

The woman having been under the care of three or four different practitioners before I saw her, I deemed it proper to examine immediately with great attention; when I found that the womb was moveable, and about as large as a goose's egg; that its mouth was broad, open, and of cartilaginous hardness; that it manifested the usual marks of malignant disorganization, in which also about one-fourth of the contiguous vagina was involved; and, further, that on the surface of the diseased mass was formed an ulcer, about as broad as a shilling. The adjacent structures appeared to be healthy enough: the bladder and rectum were sound; the inguinal glands were not enlarged, whence it was presumed that the lumbars were perhaps healthy; the ovaries could not be felt to exceed their ordinary bulk; and there evidently was no tangible enlargement of the liver, spleen, kidneys, or omentum, all of which were examined with the nicest care. The breathing was easy; the pulse, various in its frequency, ranged between 115 and 120 in the minute; and the patient, though certainly very much debilitated, had sufficient remains of strength to walk to my house, (the distance of a furlong,) though not without considerable difficulty. To be short, it seemed clear at this time that the case was ulcerated carcinoma of the uterus, as it is called, and that extirpation was the only remaining remedy.

The bowels having been cleared, and the patient being resolved to submit to the operation, on the 19th of February, 1828, I determined to remove the diseased parts without further delay. For this purpose, having placed the woman in the obstetric position usual in this country, (on the left side, I mean,) close upon the edge of the bed, with the loins posteriorly, the shoulders advanced, the knees and bosom mutually approximated, and the abdomen directed a little downwards towards the bed, I began the operation.

*First stage of the operation.*—I commenced by passing the index and second finger of the left hand to the line of union between the indurated and healthy portions of the vagina; the finger being converted into a cutting instrument, (varying with the exigencies of the operation,) by means of a moveable knife, which requires a word or two of description. The blade of this knife, not unlike that of a dissecting scalpel, was mounted upon a long slender shank, which, including its large handle, was about eleven inches in length; and with this stem the blade was united, so that its flat, or plane, formed with the stem an angle of fifteen or twenty degrees. The first and second fingers of the left hand then being in the back of the vagina, contiguous to the diseased mass, (as before observed,) by taking the stem-knife in my right hand, I could at pleasure lay the flat of the blade upon the front of these fingers, and urge the point of the instrument a little beyond the tip. The apex of the forefinger being in this manner converted into a cutting point, by little and little I gradually worked my way through the back of the vagina, toward the front of the rectum, so as to enter the recto-vaginal portion of the peritoneal cavity, frequently withdrawing the stem-scalpel, so as to place the point within the tip of the finger, and then making examination with great nicety, in order to ascertain whether the vagina was completely perforated, minute care being necessary in this part of the operation to avoid wounding the front of the intestine.

*Second stage of the operation.*—A small aperture having been formed in this manner in the back of the vagina, through this opening the first joint of the forefinger was passed, so as to enlarge it a little by dilatation and slight laceration, (safer than incision.) This done, and a cutting edge being communicated to the finger, by placing the plane of the blade in such a manner that its incisory edge lay slightly advanced beyond the side of the finger now lying in the aperture, after drawing the point of the instrument within the tip of the finger, which operated as a guard, I proceeded to make an incision through the vagina transversely,—that is, in a direction from hip to hip; for this purpose carrying the finger with its cutting edge, from the opening in the vagina already made, to the root of the broad ligament on the left side, so as to make one large aperture. I then took a second stem-scalpel, formed on the same model as the preceding, with this difference, that the incisory edge lay on the other side of the blade; and laying this instrument on the fore-finger as before,—in such a manner, however, that the cutting edge lay forth on the other side of the finger, (to the right of the pelvis, I mean,)—I carried the finger thus armed from the middle of the vagina, where the former incision commenced, to the root of the broad ligament on the right side; so that, at the end of this, which was the second step of the operation, the diseased and healthy portions of the vagina behind became completely detached from each other by a transverse incision, which stretched across the vagina, between the roots of the broad ligaments immediately below the diseased parts. At this time the intestines could be felt hanging about the tips of the fingers; but the blade of the scalpel lying on the finger, in which it was as it were imbedded, the risk of a wound, whether by point or edge, was completely prevented.

*Third stage of the operation.*—The back of the vagina, then, having been divided in this manner, I urged the whole of the left hand, not of large size, into the vaginal cavity, and the more easily because the woman had borne children; afterwards passing the first and second fingers through the transverse opening along the back of the uterus,—this viscus lying, as usual, near the brim of the pelvis, with its mouth backward, its fundus forward, and a little elevated just above the symphysis pubis. This manœuvre premised, under full protection of these fingers, now lying between the womb and the intestine, taking a double hook, mounted on a stem eleven inches long, I passed it into the abdominal cavity, through the transverse aperture, along the surface of the fingers already mentioned; and laying it in front of them, near their tips, I converted these fingers into a sort of sentient tenaculum, which, with little pain to the patient, I pushed into the back of the womb, near the fundus, and then drawing the womb downward and backward, towards the point of the os cocygis, as I carried the fingers upward and forward, I succeeded ultimately in placing the tips over the fundus in the manner of a blunt hook; after which, by a movement of retroversion, the womb was very speedily brought downwards and backwards into the palm of the left hand, then lodging in the vagina, where, at this part of the operation, the diseased mass might be seen distinctly enough lying just within the genital fissure.

*Fourth stage of the operation.*—The process of removal being brought to this point, the diseased structure, still in the palm of my hand, remained in connexion with the sides of the pelvis, by means of the fallopian tubes and broad ligaments, and with the bladder by means of the peritoneum, the front of the vagina, and interposed cellular web,—parts which were easily divided, so as to liberate the mass to be removed. The broad ligaments were cut through, close upon the sides of the uterus; and, in dividing the vagina, great care was taken to keep clear of the neck of the bladder and the ureters. This division of these attachments, and the removal of the diseased mass, constituted the fourth step of the operation. Some bits of indurated vagina, altogether not larger than the common bean, were left in the pelvis, to be removed at some future period, should symptoms require. This fact is worth recording.

To this circumstantial account of the operation may be added a few remarks. The intestines did not protrude. About an ounce of blood was lost when the back of the vagina was divided, three or four more ounces following when the vagina was cut in front. Ligatures, tenacula, and forceps, were in readiness to secure the vessels, but these were not required.

The intestines were felt at one time only, namely, when two fingers were lying out through the opening in the vagina behind. Of course, some pain was felt when the first incisions were making, and when, as in ordinary obstetric operations, the hand was urged into the vagina; but the principal distress was occasioned by drawing down the uterus, when the retroversion was accomplished, and the ligaments were put upon the stretch.

The pains and complaints scarcely exceeded those observed in instrumental deliveries. The patient lay in the ordinary obstetric position, and required no restraint. The insertion of the hook into the back of the uterus did not occasion much suffering. The operation, from first to last, occupied about an hour; but much of this time was spent in reposing, and considering what might best be done. With better instruments, and greater activity, the whole operation might most probably be completed in five minutes. In obstetrics, however, celerity is considered to be in itself a secondary merit, and the operation was conducted on obstetric principles. The general range of the pulse was between 120 and 130, a frequency common in delivery by instruments.

When the last gush of blood was observed, the pulse became imperceptible in the wrist; returning, however, in the course of ten or fifteen minutes. A few ounces of spirit were administered to the patient as the operation proceeded. Throughout the process the forefinger of the left hand was the principal instrument, and the scalpels and hooks were employed merely as the means of arming the finger for its various operations.

The professional friends who favored me with their presence were, Dr. Elliotson, Mr. Callaway, Mr. B. Cooper, Mr. Key, and Mr. Morgan. An accident deprived me of the presence and assistance of my friend Dr. Roots.

The operation was not undertaken at a venture, but in conformity with certain principles laid down in two papers read before the Medico-Chirurgical Society; the first of them in the year 1819, and the last in the year 1823. The latter, which was not published, contains the proposals for other abdominal operations. The fundamental principles of these operations, as there stated, are rested upon numerous observations made upon the human body, and a sufficient number of experiments upon brutes. Should the case here narrated come before the eyes of the public, I hope it may tend to diminish any unreasonable prejudices against experiments and experimenters. The feeling is respectable, but by the designing it may be misdirected. In Lisfranc's operation I conceive there must be some misapprehension. I think I run no risk in saying that by his method of procedure, as understood here, what the English accoucheur means by cancer of the uterus must frequently be irremovable.

It is now five months since the parts were extirpated, and the patient is fat and well, and designs to return to her husband. The interception of the access to the ovaries is a complete security against extra-uterine impregnation. The head of the vagina is closed by the bladder, which lies upon it. The recovery was easy enough, but, as the details may perhaps be deemed desirable, they shall be communicated at an early opportunity. The patient had been ill for eight or nine months before the operation was performed.—*Medical Gazette.*

---

*On the Treatment of Ulcers by Plates of Lead.* By Dr. A. MENON.—In all wounds, the simpler the treatment the better. This principle of surgery is now so generally recognised, that there remain few practitioners who have not laid aside all multiform and compound applications to sores, and which only retard their cure. Quiet, cleanliness, dressings with lint, either dry or spread with salve, form the basis of treatment now followed, unless in very particular cases. M. REVEILLE PARISE has suggested a still simpler mode of treatment. In a Memoir read some time since at the Academie de Medicine, he states that he had derived the most advantageous results from plates of lead applied to wounds. GUY DE CHAULIAC and AMBROSE PARÉ had before employed, in the cure of ulcers, plates of lead rubbed with mercury; but this means, neglected by men of science, passed into the hands of the vulgar, where it is still occasionally met with. The following fact will show this.

A military invalid had applied to his ulcers a plate of lead: surprised to observe that they regarded his being dressed by this means as a new fact, he said that, twenty years before, having received a wound on the field of battle, he cured himself by applying a leaden bullet that he beat out with a flint. He said that this remedy was known to many soldiers, who always tried it when their wounds were not sufficiently bad to go into hospital.

M. PARISE, however, has not the less merit in restoring to science a mode of cure which may be very useful. Recent wounds are almost the only ones in which he has tried his plan. His success induced M. le Baron YVANS to repeat the trials; and the great number of people affected with ulcers in the establishment at the head of which he is, gave him great facility to make the experiment.

M. MENON has not seen a sufficient number of recent wounds tried with this means, to speak of its effect; but more than sixty cases of ulcers successfully treated in this way convince him that the plates of lead form the best topical application to ulcers. Among these cases, some have been of thirty years' standing. When they were not completely cured, the ulcer quickly put on a new aspect: its edges thickened, diminished in size, and soon assumed a more satisfactory appearance. It was remarked that several ulcers, which had never been able to be cicatrised, were so by this new method; and that those in which it failed were never cured by any other means: and we may conclude that these last were incurable, and that nature, more powerful than art, resisted the suppression of an evacuation that custom had rendered necessary. At first this method was confined to simple ulcers: emboldened by success, M. Yvans tried it on ulcers evidently in an inflammatory state, and which before he would have treated by topical emollients. He was astonished to see that the lead not only did not increase the pain, but the inflammatory symptoms diminished, and shortly the ulcer was in a healthy state. In fact, many cases prove that this substance acts with a promptitude quite surprising in bringing ulcers foul at bottom, and having all those appearances that are implied by the name "ulceres sordides," back to a healthy state. Three or four days has in general been sufficient, and soon after cicatrization has generally begun to take place.

The fact being established that lead, in the form of plates, applied to wounds is beneficial, it would be interesting to know how it acts. Does this lead enjoy any specific property? Can it have within it something peculiar which, changing the kind of irritation of the ulcerated surface, disposes it to heal? Is there any chemical action takes place, from which might result a compound analogous to the acetate of lead which GOULARD and others have so much praised? M. Menon thinks not; for it appears that plates of brass, or gold, or silver, have nearly the same results. Lead is, however, preferable, both being cheaper and also more flexible, so that it is modelled more exactly to the surface. M. Menon thinks it acts mechanically, exercising slight compression on the borders of the wound, and keeping in contact with it the pus which many physiologists think necessary to the formation of a cicatrix.

This mode of dressing consists in the simple application of a thin plate of lead on the ulcer, and keeping it in its place by a bandage. When it is wished to be removed, the surface of the ulcer and the plate are both wiped clean, and again brought into contact. Nothing can be simpler; and it is a great advantage, not exposing the surface of the sore too long to the action of the air. If granulations shoot up too fast, touch them with lunar caustic, or powder them with calcined alum.—*Archives generales de Medicine.*

---

*On Sanguineous Tumors of an equivocal character, which appear to be Aneurisms of the Arteries of Bones.* By M. BRESCHET.—These M. Breschet thinks have been confounded by writers with osteo sarcoma, fungous hæmatodes, inflammation of the veins of bones, and of the bones themselves, &c. The disease generally arises spontaneously, sometimes follows blows or other external violence, occasionally succeeds gouty or rheumatic affections of the knee. It is usually situated at the posterior part of the leg, below the knee, and affects the tibia or fibula, or both; sometimes the disease occupies the metatarsal bones of the foot. The tumor is painful, and the veins of the whole limb swollen, distended, and varicose. There is deep-seated pulsation, synchronous with that of the arteries: this pulsation ceases on the artery, between the heart and tumor, being compressed. On pressing with the finger upon some parts of the tumor, a sound resembling the breaking of an egg may be heard. On dissection, the cellular tissue of the bones has always been found in great part or wholly destroyed; the cavity of the bone enlarged, filled with coagulated blood disposed in concentric layers, as in old aneurismal tumors, and these clots form one or several foci, each communicating with an arterial branch. The external table of the bone still remains, but thinner than natural; destroyed in several of its parts, and offering but feeble resistance, in comparison with a cartilaginous surface, which yields to the finger, but quickly recovers itself. Sometimes the bone may be crushed easily, like an egg-shell. The periosteum and the aponeurotic expansion are generally thicker and firmer than in the healthy state, and sometimes they pass into a fibro-cartilaginous state. The articulation near the seat of the disease has always been found healthy, even when it was only separated from the seat of the malady by an expansion of cartilage. Injection has proved that the principal vessels of the limb are healthy through their whole extent: this is not the case, however, with the small arteries which supply the substance of the bone: they are increased in size, and pass from the centre of the bone into the aneurismal sac by several orifices.

M. Breschet thinks that this affection may be compared to erectile tissues of soft parts, and that the pulsation in them, which is sufficiently powerful to have led to the comparison between them and aneurisms, properly so called, results from the synchronous movements of dilatation and contraction of all the small arteries which pass from the bony substance to the parts affected. From these partial but simultaneous movements results a continued motion, which has also been frequently observed in erectile tumors of the lips, and of the globe of the eye, &c. That the disease is of an aneurismal character, is proved by the fact that the ligature of the principal trunk arrests the disease.

The credit of being the first to detect the nature of this disease, and also of having first indicated the best mode of treatment, is due to DUPUYTREN.

M. Breschet is of opinion that neither topical applications nor compression can be of any service in this affection; the ligature is the proper remedy. The sooner, however, it is applied, the greater the chance of success; for the farther the disorganization of the osseous tissue has advanced, the greater will be the difficulty of cure, even when the aneurismal character of the complaint is removed by the ligature. When considerable disorganization has taken place, amputation offers the only hope of a cure.—*Repertoire général d'Anatomie, &c.*

---

*Two Cases of Injury of the Head, accompanied with a loss of Brain.* By THOMAS SEWALL, M.D. Professor of Anatomy and Physiology in the Columbian College, D.C.

CASE I.—In February, 1827, I was called to W. Brown, a coloured man, aged fifty years, who, in a rencountre with another individual, had received a severe blow on the right side of the head with a sharp spade. When I arrived, which was only a few minutes after the accident, I found him bleeding profusely, and already so much exhausted from the loss of blood as scarcely to be able to support himself. Though not insensible, he had lost his reason, and appeared not to know how he came by the injury. On examination, I found a deep wound dividing the integuments, the whole of the temporal muscle penetrating the cavity of the cranium, and extending horizontally, from an inch above the external angular process of the frontal bone, through the parietal bone just above the squamous suture, forming a fissure of three inches in length. The lower portion of bone was considerably depressed, and the two edges separated about half an inch.

Two branches of the temporal artery were taken up; when, on a more critical examination, it was ascertained that the dura mater was divided for an inch in extent, and the brain penetrated some way into its medullary portion, which was easily distinguished from its cortical part.

Suitable dressings were applied, and he was conveyed home, about one mile distant, and placed in bed with his head and shoulders considerably elevated. From the great loss of blood, his pulse was feeble, and his extremities cold. Warmth was applied to the limbs: he soon became sensible, and complained of severe pain in the head and vertigo. The most rigid antiphlogistic course was enjoined, and the patient placed under the immediate care of an intelligent student, who was directed to bleed and to purge in proportion to the reaction of the system, and with a freedom that should prevent any bad effects from subsequent inflammation. He was bled and purged daily for a considerable time, the circulation equalised by warmth applied to the extremities, and by gentle diaphoretic remedies given internally.

During the process of suppuration, the brain protruded and sloughed away, and subsequently portions were removed by a spatula.

A few days after the accident, a second wound was discovered, which penetrated the integuments and the frontal near the median line, and about one inch from the coronal suture. This wound was apparently made by a small spear-pointed instrument, and was so large as to admit a probe to pass through the skull.

For about ten days after the accident, the patient complained of constant and sometimes of severe pain in the head; and on one occasion was affected with a slight spasm of the muscles of the face, neck, and extremities. The wound healed, and in six weeks the patient was quite well. He has since followed his occupation, that of scavenger, and has not manifested any deviation in the functions either of body or mind from their ordinary healthy condition.

Case II.—September 18th, 1827, Lewis Pool, aged five years, while playing in the street, was kicked by a horse, and taken up in a state of insensibility. I arrived a quarter of an hour after the accident, and found a semicircular wound in the integuments of the head, and, corresponding with this, a large fissure in the frontal and parietal bones, about three inches above the external angle of the right eye. Through this fissure a portion of brain protruded, something larger than a walnut, and was composed both of cortical and medullary matter, which were easily distinguished. This was so far separated from the parts beneath as to be removed without any violence.

Light dressings were applied, and the patient placed in bed. The pulse was slow and intermitting, the pupil dilated, and the skin cold. Very little blood had been lost from the accident. In a few hours he became sensible; the pulse rose, and he complained of pain in the head. He was bled to fainting.

Particular circumstances prevented the subsequent use of the lancet; but he was purged actively and daily for two weeks, and the pulse kept down by nauseating doses of the tartrate of antimony. Extensive suppuration came on, with a copious discharge of pus; the wound gradually healed; and in about five weeks the child was quite well. He has since remained in perfect health. —*American Journal of Medical Sciences.*

---

### CHEMISTRY.

*A Method of detecting the Presence of Opium in very small Quantities.*—Dr. R. Hare, of Pennsylvania, has discovered a process by which the presence of opium contained in a liquid in the proportion of twelve drops of laudanum to half a gallon of water, may be detected. This method is founded upon the property which the meconic acid possesses of forming a precipitate with the solutions of lead. Thus, if a few drops of acetate of lead are dropped in an infusion containing opium in as small a proportion as above mentioned, an evident precipitate of meconiate of lead is obtained. If the quantity of opium is small, the precipitation is not effected in less than from six to twelve hours. It may be forwarded by being gently stirred with a glass tube, to detach the flakes from the sides of the vessel, which should be of a conical shape to facilitate their approximation during precipitation. When the meconiate of lead is thus settled at the bottom of the vessel, thirty drops of sulphuric acid must be poured into it by means of a glass tube, added to which must be an equal quantity of the red sulphate of iron. The sulphuric acid, uniting with the lead, sets free the meconic acid, which, by combining with the iron, produces that particular red colour indicative of the presence of that acid, and consequently of opium.

---

*Gallates of Quina and Cinchonia.*—M. PLATANIA forms these compounds in the following way: Pour an infusion of galls into a hot solution of the sulphate of quina, wash the precipitate with cold water upon a filter, and dry it at 100° of Fahr. Afterwards dissolve it in warm alcohol; pour off the solution, and evaporate it; then add cold water to it, which precipitates pure gallate of quina.

Another process consists in pouring gallic acid into sulphate of quina, and merely washing the precipitate with cold water; and it may also be formed by directly combining the acid and base, each separately dissolved in alcohol. Gallate of quina is very white and light; its specific gravity is 0.816 at 60° Fah. Its vapour is astringent, and very slightly bitter; it is soluble in alcohol and ether, but almost insoluble in water. It is composed of nearly

| | |
|---|---|
| Gallic acid ............ | 14.87 |
| Quina ................. | 85.13 |
| | 100.00 |

The gallate of cinchonia is obtained by dropping gallic acid into a solution of cinchonia; the gallate precipitates, and is to be redissolved in water, and suffered to cool; the liquid becomes opalescent, and deposits granular transparent crystals.—HENSMAN's *Repertoire de Chimie, &c.*

---

*Action of Alkalies, and their Carbonates, &c. on Iodides.*—M. BERTHEMOT, having made numerous experiments on the action of alkalies and some metals on the iodides, concludes: That the earthy oxides, and their carbonates, do not act upon iodide of mercury;—that potash, soda, barytes, and strontia, decompose iodide of mercury by the intervention of water or alcohol, and there result oxide of mercury and tri-iodo-hydrargyrate of potash, which, on the cooling of the liquors, successively deposit iodide of mercury and bi-iodo-hydrargyrate of potash;—that lime produces the same phenomena, with this difference, however, that the action occurs only by the intervention of alcohol;—that the soluble carbonates of the alkaline oxides also decompose iodide of mercury, and yield analogous products, but only with the intervention of alcohol;—that the insoluble carbonates of the alkaline oxides do not act upon iodide of mercury, either by the intervention of water or alcohol;—that the protoxide of mercury decomposes the iodide of potassium, forming potash and metallic mercury, or protiodide of mercury and iodo-hydrargyrate of potash;—that the remaining alkaline iodides have a similar action, except that of calcium, which does not appear susceptible of it;—that peroxide of mercury decomposes the alkaline iodides, forming an alkaline oxide and bi-iodo-hydrargyrate.—*Journal de Pharmacie.*

---

### MISCELLANEOUS.

*Cupping Glasses to Poisoned Wounds.*—Dr. PENNOCK, of Philadelphia, has lately repeated a series of experiments similar to those which had been previously made by Dr. BURRY. The conclusions Dr. P. has arrived at are as follow: viz.

1

1st. The usual effects of poisoned wounds cannot take place during the absence of the atmospheric pressure procured by the application of cupping glasses.

2d. Such application does not arrest the deleterious action of the poison by withdrawing it from the exposed surface. On the contrary, the fatal effects are wholly prevented, though not a particle of the substance employed has been abstracted. In proof of this, if a poison in powder (strychnine or arsenic, for instance,) be conveyed by a tube through a narrow wound, in an oblique direction under the integuments, to some distance from the opening by which it is introduced, and there deposited, and under these circumstances the glass be applied over this spot, where the skin is sound and unbroken, the wound being without the bounds of the glass, none of the poisonous substance will be removed, and yet no indication of its action will be presented during the time of the application of the glass.

3. The constitutional symptoms, such as tetanic convulsions, &c. are arrested by the establishment of a vacuum on the poisoned surface; then, by removing the poison by an incision through the integuments, the life of the animal is preserved.

4. When the cupping glass is applied over the opening made in the integuments, for the purpose of introducing the tube containing the poison, and this is deposited under the skin beyond the circumference of the glass, none of the effects are manifested during the continuance of the vacuum; but, as soon as the cup is removed, the action of the deleterious article commences.

5. If, during the application of the cupping glass, placed as just stated, an incision be made between its edge, and the place at which the poison has been lodged, death will ensue as speedily as though the atmospheric pressure had not been removed.

6. If, after the application of the glass, for a given time, to the sound skin over the spot where the poison has been deposited, the glass be removed, *death will then ensue as soon as if no such application had been made.*

This last position is entirely at variance with the observations of Dr. Barry. He expressly says, that, "after the glass had been taken off, the animal continued for one or two hours to carry imbedded in his cellular tissue a dose which would infallibly have destroyed him in a few minutes, had the cupping glass not been previously applied." Dr. Pennock has, however, repeatedly observed that if the animal was abandoned to his fate after the glass had been removed, after an application of it for an hour or more, that death took place as soon afterwards as it ordinarily did when no vacuum had been formed. This very important practical fact has been verified by numerous experiments.

Being desirous of rendering more generally useful the method of preventing the fatal effects of poison by the exhaustion of cupping glasses, Dr. Pennock resolved to institute a series of experiments on the bite of the rattle-snake. He did not succeed, however, in procuring one of these animals whose bite was mortal. The difference of the habits of the animal whilst in captivity from those in a state of nature, and the want of a proper subsistence for several months, probably produce inertness of the secretion. The supposed specifics for the bite of a rattle-snake owe their reputation to having been used where no fatal effects would have resulted.—*American Journal of Medical Sciences.*

